# Comparative analysis of human and mouse transcriptomes of Th17 cell priming

**DOI:** 10.18632/oncotarget.7963

**Published:** 2016-03-07

**Authors:** Soile Tuomela, Sini Rautio, Helena Ahlfors, Viveka Öling, Verna Salo, Ubaid Ullah, Zhi Chen, Saara Hämälistö, Subhash K. Tripathi, Tarmo Äijö, Omid Rasool, Hayssam Soueidan, Lodewyk Wessels, Brigitta Stockinger, Harri Lähdesmäki, Riitta Lahesmaa

**Affiliations:** ^1^ Turku Centre for Biotechnology, University of Turku and Åbo Akademi University, Turku, Finland; ^2^ Department of Computer Science, Aalto University, Espoo, Finland; ^3^ Division of Molecular Immunology, MRC National Institute for Medical Research, London, United Kingdom; ^4^ Computational Cancer Biology, Division of Molecular Carcinogenesis, Netherlands Cancer Institute, Amsterdam, The Netherlands

**Keywords:** Th17 cell priming, RNA-seq, comparative analysis of human and mouse, lncRNA, disease-associated SNPs, Immunology and Microbiology Section, Immune response, Immunity

## Abstract

Uncontrolled Th17 cell activity is associated with cancer and autoimmune and inflammatory diseases. To validate the potential relevance of mouse models of targeting the Th17 pathway in human diseases we used RNA sequencing to compare the expression of coding and non-coding transcripts during the priming of Th17 cell differentiation in both human and mouse. In addition to already known targets, several transcripts not previously linked to Th17 cell polarization were found in both species. Moreover, a considerable number of human-specific long non-coding RNAs were identified that responded to cytokines stimulating Th17 cell differentiation. We integrated our transcriptomics data with known disease-associated polymorphisms and show that conserved regulation pinpoints genes that are relevant to Th17 cell-mediated human diseases and that can be modelled in mouse. Substantial differences observed in non-coding transcriptomes between the two species as well as increased overlap between Th17 cell-specific gene expression and disease-associated polymorphisms underline the need of parallel analysis of human and mouse models. Comprehensive analysis of genes regulated during Th17 cell priming and their classification to conserved and non-conserved between human and mouse facilitates translational research, pointing out which candidate targets identified in human are worth studying by using *in vivo* mouse models.

## INTRODUCTION

Th17 cells are a IL17 secreting subset of CD4+ cells and deficiency of their function leads to susceptibility to extracellular bacterial and fungal infections [1]. Moreover, Th17 cells contribute to pathogenesis of inflammatory and autoimmune diseases such as asthma, rheumatoid arthritis, psoriasis and multiple sclerosis [2]. Th17 cells play also a context-dependent role in cancer biology and can either contribute to immunosurveillance or promote malignant growth [3]. The naïve CD4+ T cell pool capable of developing into Th17 cells upon appropriate signals is maintained by thymic output and peripheral proliferation [4]. Thus, one alternative for therapeutic intervention is targeted modification of the differentiation process requiring knowledge of factors needed for Th17 cell polarization. In this study, we aimed at identifying novel factors regulating human Th17 cell polarization as well as shared and species-specific Th17 cell signatures between human and mouse by exploiting time series RNA sequencing (RNA-seq) data.

The coding transcriptome is complemented with a variety of structurally and functionally different non-coding RNA species [5]. Long non-coding RNAs (lncRNA) have been reported to have a higher tissue and species-specific expression pattern than protein-coding genes. In fact, a substantial proportion of lncRNAs identified by ENCODE project were shown to be primate-specific [6]. The studies have mainly concentrated on the analysis of a subgroup of lncRNAs called long intergenic RNAs (lincRNA) [7]. A lincRNome analysis of mouse T cell development and differentiation revealed also a group of Th17 cell-specific lincRNAs [8]. In human, peripheral blood Th subtypes and *in vitro* polarized Th cells, including Th17 cells, are reported to express lineage-defining lncRNAs [9, 10, 11, 12]. In our recent study we found that lncRNAs mapping to loci shared between various immune-mediated diseases were significantly enriched in immune cell types compared to lncRNAs from the whole genome [10]. In this study, the lncRNAs differentially regulated during human Th17 cell priming were identified for the first time.

Systems biology approaches have been exploited to characterize transcriptional regulation during Th17 cell differentiation in mouse [13, 14]. However, comparison of the Th17 cell differentiation process in model organisms and in human is missing. In this study, the top 20% of the differentially expressed genes were ranked and the results between human and mouse compared. Using these strongly regulated genes, altogether 307 genes were found to be regulated similarly in both human and mouse Th17 cell priming at least at one time point. The expression profiles and levels of Th17 cell-specific coding transcripts were analysed to reveal the level of conservation in the gene expression patterns. The data was also used to predict the key transcriptional regulators and co-ordinately controlled Ensembl genes. Finally, the genes identified to belong to the Th17 cell-specific transcriptome were overlaid with the single nucleotide polymorphisms (SNP) known to be associated with human diseases. Our results indicated that identification of similarly regulated genes between human and mouse pinpoints signaling pathways predisposing to diseases, which can be studied with mouse models. In addition, species-specific differences, which could be due to both biological and technical reasons, dominate especially among the long non-coding transcripts.

## RESULTS

### Gene expression in human Th17 cell induction

**Figure 1:**
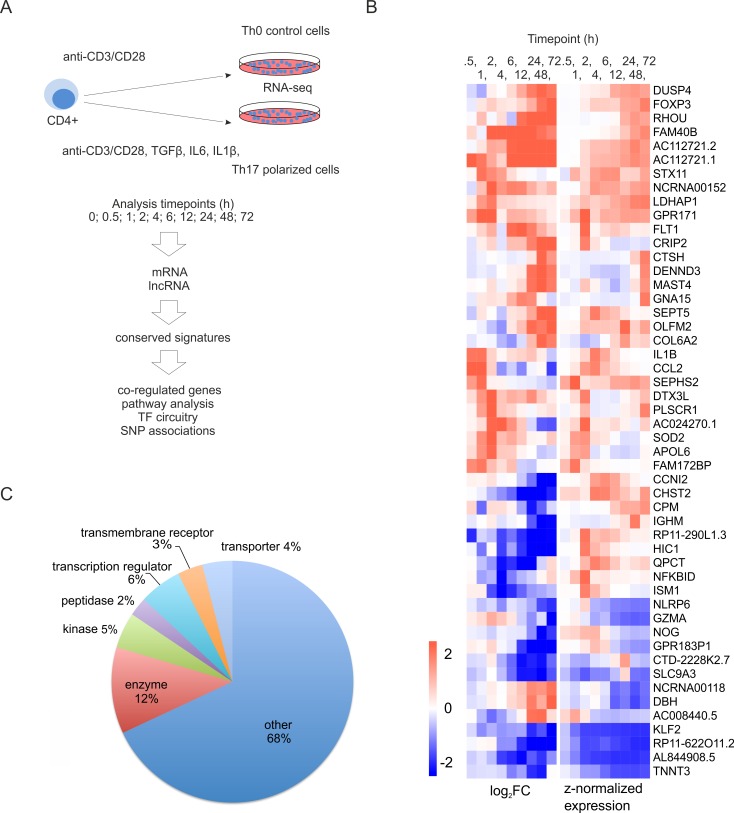
Transcriptional changes during the first 72 hours of Th17 cell differentiation **A.** Schematic overview of the approach used in the study. CD4+ cells were cultured under Th17 cell polarization condition. Three biological replicates of the time-series were collected for RNA-seq. **B.** Heatmap of the selected human genes associated with Th17 cell polarization for the first time in this study. The visualized genes were differentially regulated between Th17 and Th0 conditions at least in two timepoints, and their expression level was more than 10 RPKM in some of the sampling timepoints. The genes were ranked based on their average absolute log2 FC over the timepoints. Top 50 genes were visualized in the heatmap, where genes were clustered using hierarchical clustering. **C.** Functional annotation (www.ingenuity.com) of the human genes not previously reported to be differentially regulated during Th17 cell polarization. The differentially expressed genes were considered as unreported if they were not indicated to be regulated during Th17 cell polarization in the previous high-throughput studies [13, 14, 15].

We exploited RNA-seq to investigate global gene expression profiles during the early Th17 cell differentiation in human, using CD4+ cells isolated from umbilical cord blood (Figure 1A). We found 2001 Ensembl genes (here after called as genes), differentially expressed specifically in the Th17 cell polarization condition compared to undifferentiated Th0 samples at least at one time point (Table S1). Out of these genes, 74% were not found to be differentially expressed in our previous microarray study on human Th17 cell differentiation [15], and 80% of these novel hits have not been reported to be differentially expressed in RNA-seq studies investigating Th17 cell priming in mouse [13, 14]. A considerable number of the genes which were found to be differentially regulated during Th17 cell differentiation for the first time in this study had a substantial expression level and magnitude of differential expression between Th17 and Th0 control cells (Figure 1B). These genes also represented several functional classes (Figure 1C).

Altogether 11% of the Th17 cell subtype-specific genes reported by Ranzani *et al*. 2015 [11] by investigating Th17 cells isolated from peripheral blood were also found to be differentially regulated in our data at some stage during Th17 cell priming (Table S1). The overlap between the gene expression profiles of Th17 cells isolated from peripheral blood [11] and our data increased toward the latest analysis timepoints as expected, being highest 7.5% at 72 hours. The genes present in both datasets were mainly Th17 cell marker genes such as *IL17*, *CCL20*, *CCR6* and *IL23R* indicating that kinetic analysis of *in vitro* differentiation of Th cells is essential for identification of novel priming factors needed for Th17 cell polarization.

### Human Th17 cell lncRNAome

**Figure 2:**
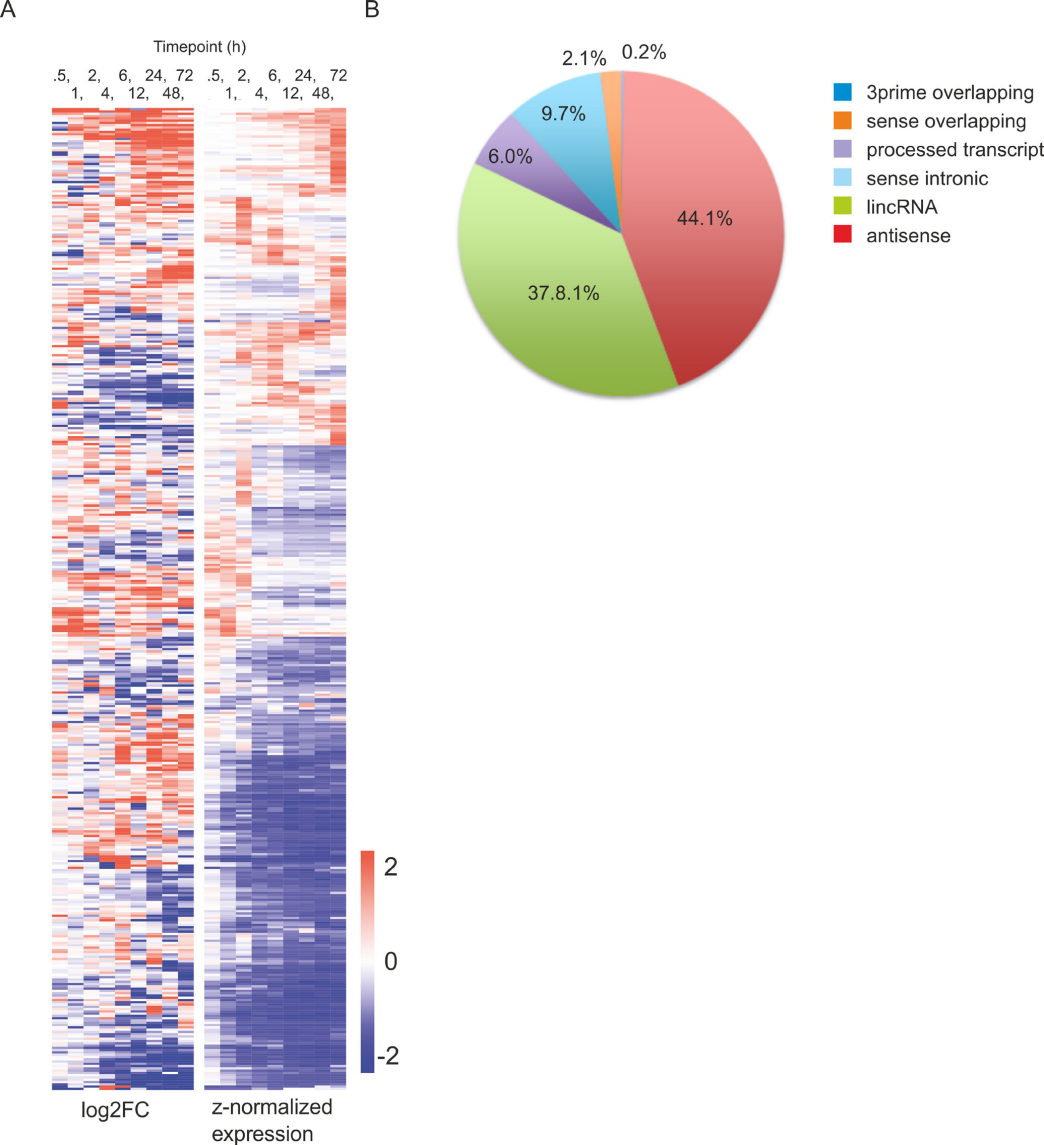
Differentially expressed lncRNAs during human Th17 cell priming **A.** Heatmap of the differentially expressed lncRNAs between cells polarized toward Th17 phenotype and unpolarized control cells (FDR <0.05, |log2 FC| >1 cut offs and RPKM >0.5). The lncRNAs were clustered using hierarchical clustering with Euclidean distance. **B.** Classification of the differentially expressed lncRNAs.

In the analysis of non-coding component of the transcriptome, we found 7368 lncRNAs to be expressed in human cord blood CD4+ cells before activation (Table S2). In general, activation resulted in dramatic downregulation of lncRNAs as altogether only 2857 lncRNAs were found to be expressed in human CD4+ cells after activation (Table S2). Out of the lncRNAs expressed after activation, 431 showed differential expression in Th17 cells (Figure 2A). The average expression level of lncRNAs did not change in response to activation, or did not differ between all lncRNAs which were expressed after activation and the ones which responded to Th17 cell polarization (Figure S1). The majority of lncRNAs were antisense transcripts or lincRNAs both representing around 40% of the differentially regulated lncRNAs identified (Figure 2B). Antisense transcripts and sense-intronic RNAs were statistically significantly (*p* < 0.005) overrepresented, and lincRNAs underrepresented (*p* < 0.005) among the differentially expressed lncRNAs in our data (Figure 2B).

Comparison of our lncRNA data with the Th17 cell denoting lncRNA profiles acquired through Th17 cell polarization among peripheral blood PBMC pool [12] or *via* isolation of Th17 cells based on their cell surface epitopes [11] revealed that the lncRNA expression profiles are highly specific to experimental set up. There was no overlap between peripheral blood Th17 cell-specific lncRNAs profiles acquired with *ex vivo* [11] and *in vitro* differentiation [12] approaches. Similarly, when the list of differentially regulated lncRNAs in our study was compared with the lncRNAs specific for peripheral blood isolated Th17 cells [11] no overlap was found. However, when our lncRNA data was overlaid with the data gathered from PBMC pool stimulated with Th17 cell polarizing cytokines [12] altogether five lncRNAs were found to be similarly upregulated, namely *RP11-98D18.3*, *AL450992.2*, *RP11-509E16.1*, *LINC00877* and *LUCAT1*. Interestingly, lncRNAs *RP11-430C7.4*, *CHRM3-AS2*, *CHRM3-AS2*, *AC007278.3*, *AC008063.2*, *LINC00861* and *CTC-378H22.2* reported to be overexpressed specifically in Th1 cells, and Th2 cell lincRNAs *RP3-395M20.8* and *RP11-234B24.4* [12] were downregulated in our Th17 cell data.

The Gene ontology (GO) enrichment analysis of the nearest coding genes and the differentially expressed genes having the highest positive or negative expression correlation with the identified Th17 cell differentiation denoting lncRNAs was used to predict the functional role of these non-coding transcripts. The differentially regulated lncRNAs were neighbors of the genes involved in e.g. T cell activation and proliferation or cytokine production (Table S3). Among the genes with the highest inverse correlation with the differentially expressed lncRNAs enrichment was also found for genes belonging to GO-classes associated with regulation of cell proliferation (Table S3).

### Regulation of Th17 cell signatures in human and mouse

**Figure 3:**
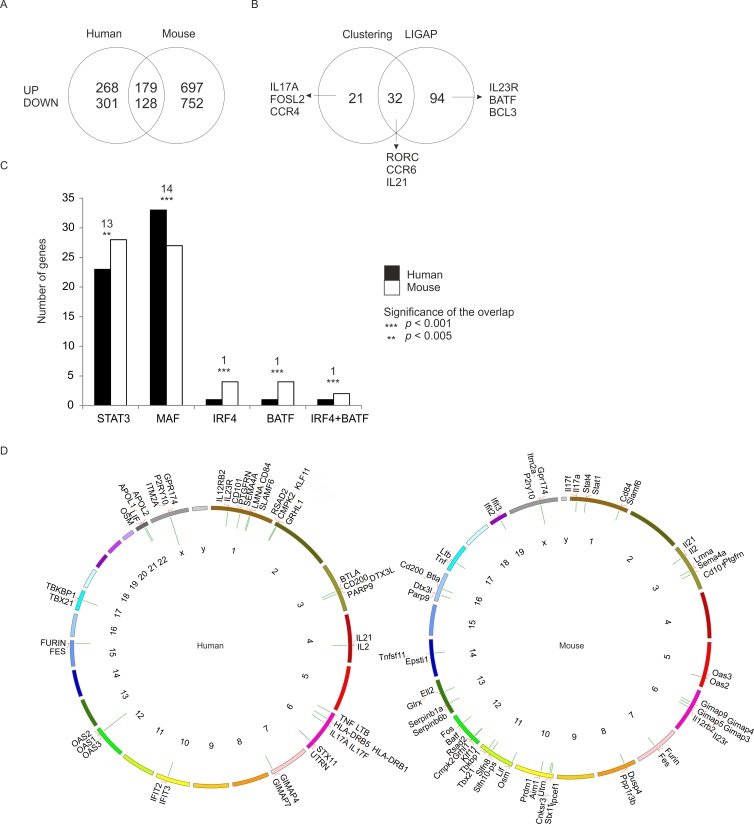
Shared Th17 cell-specific transcriptome in human and mouse Genes with FDR <0.05 were ranked based on their fold change between Th17 and Th0 conditions. The top 20% of the ranked up-regulated and down-regulated genes were included in the analysis. **A.** Comparison of the regulation of the orthologous genes. The similarly regulated genes had conserved regulation at least at one time point during the analyzed time frame (UP/DOWN = up- / down-regulated genes in Th17 cells). **B.** Comparison of the clustering and the time shift analysis results. Clustering analysis was used to select the orthologs which shared their expression profile in both species as judged by their presence in the same cluster when clustering was performed over standardized time profiles averaged at each time point across replicates. Orthologous gene pairs with similar time-shifted profiles in their Th17 cell expression were determined with an extended LIGAP method [16]. **C.** The number of genes predicted to be bound by STAT3, MAF, IRF4, BATF or IRF4 and BATF together based on the comparison of our data with the data by Ciofani *et al.* 2012 [13] with analysis window of +/−250 bp around the transcription start sites (TSS). The number of the similarly regulated genes between human and mouse that have the same binding motif is indicated above the bars with the statistical significance of the overlap. **D.** Genes similarly regulated in human and mouse (Table S4) were clustered based on their chromosomal location. The clusters of co-localizing genes were identified and the clusters with statistically significant (*p* < 0.05) co-localization visualized with the Kerfuffle tool [35] for human and mouse. The green bars protruding inward in the Circos plots indicate the identified clusters and the length of the bars represent the numbers of genes in each cluster.

The kinetic expression profiling was replicated with naïve CD4+ cells isolated from spleens and lymph nodes of C57BL/6 mice to further highlight the genes which characterize initiation of human Th17 cell development (Figure 1A). In mouse, altogether 4052 genes were found to be differentially expressed in Th17 cells compared to Th0 cells (Table S1). The genes with FDR <0.05 between Th17 and Th0 cells were ranked based on their fold change and the top 20% of the ranked up-regulated and down-regulated genes at each time point were used in the interspecies comparison. Altogether, we identified 307 human and mouse orthologous gene pairs that were similarly regulated in both species at least at one time point (Figure 3A, Figure S2A, Table S4). The most strongly regulated, i.e. the top 20% of the genes, included 44% and 54% genes not linked to Th17 cell differentiation in previous mouse and human profiling studies, respectively [13, 14, 15]. On the functional level, cytokine genes and genes related to regulation of transcription (transcription factors and ligand-dependent nuclear receptors) were significantly enriched among the top 20% of human and mouse genes (Table S5). The same phenomenon was observed at the signalling network level as Gene Set Enrichment Analysis revealed several cytokine or chemokine pathways, and pathways involved in regulation of transcription to be enriched in both species (Figure S2B, Table S6).

To identify the genes which share their whole expression profile the differentially expressed human and mouse genes were clustered together. Altogether 53 gene pairs e.g. *IL17A*, *CCR4* and *FOSL* were recognized to respond similarly to Th17 cell induction with this method (Table S7). A possible time difference in Th17 cell expression profiles among the shared differentially expressed genes in human and mouse was taken into account using a time shift parameter extension to the LIGAP method [16] (see Supplementary Information) revealing similar behaviour in 126 gene pairs (Figure 3B, Figure S3, Table S7). Comparison of the expression level of the differentially expressed genes declared that in general the expression levels of Th17 cell-specific genes are positively correlated (range> 0.59-0.76) between the species (Figure S3, Table S7). This indicates that cooperative and synergistic functions could be conserved between human and mouse. However, it should be noted that over 50% of the top Th17 polarization denoting genes observed in human and mouse did not have comparable overall expression pattern based on LIGAP and clustering analyses (Figure 3A, Figure 3B).

To further correlate the transcriptional circuitry in human and mouse, we compared our data with the reported BATF, IRF4, STAT3, MAF, and RORC chromatin binding patterns at 48 hours after initiation of mouse Th17 cell differentiation [13]. Enrichment for STAT3, MAF, BATF, IRF4 and composite IRF4+BATF binding motifs [13] in promoter-proximal chromatin binding sites, and statistically significant overlap of predicted target genes between the species was found (Figure 3C, Figure S4, Table S8). Thus, our data is consistent with the previous report indicating that these factors form the core of Th17 cell-defining transcriptional regulation [13], here shown to apply to both mouse and human. In addition, we searched for differentially regulated genes which shared chromosomal location and thus could be under regional co-regulation. In human 21 clusters and 29 in mouse contained genes, which were statistically (*p* < 0.05) localized closer to each other than could be expected by chance (Figure 3D, Table S9). Conserved clustering suggests that these genes are under evolutionary pressure to preserve coordinated regulation. For example, adjacent location of the genes coding for cytokine receptors IL23R and IL12RB2, has been suggested to enable switching between Th17 and Th1 cell promoting receptor expression pattern *via* competitive usage of transcriptional regulatory sites [17].

As orthologous relationships among lncRNAs are not comprehensively known, we used an alternative approach to compare human and mouse lncRNomes. By converting the human lncRNA coordinates into mouse coordinates [18] we identified only 25 lncRNA pairs similarly regulated in a Th17 cell-specific manner in human and mouse (Table S2). Moreover, a sequence level comparison revealed that less than 40% of the lncRNAs expressed in human cells during Th17 cell priming had at least 50% sequence similarity even in the mouse genome (Figure S5).

### Overlay of transcriptome to disease-associated polymorphisms

**Figure 4:**
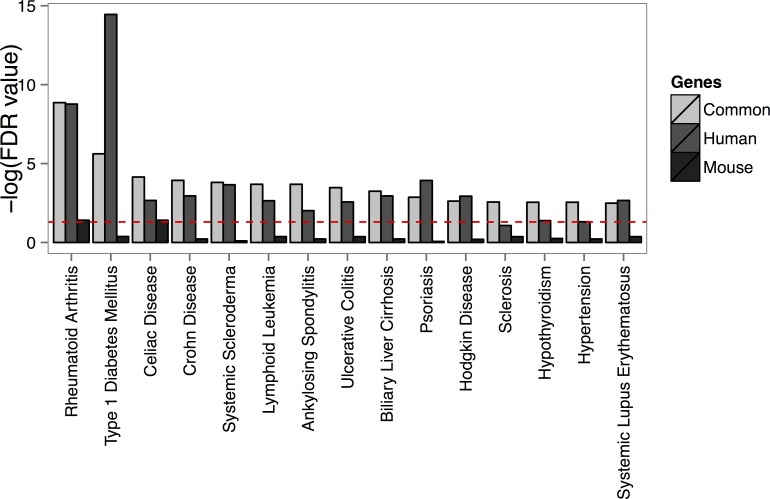
Disease-associated single nucleotide polymorphisms are localized close to the identified Th17 cell-specific genes The enrichment of the known lead SNPs associated with diseases among the orthologous genes differentially regulated in Th17 cells are presented in the figure for the shared Th17 cell-specific genes between human and mouse (common), and the human and mouse top 20% coding transcripts. The figure summarizes the data for 15 diseases with the highest enrichment of SNPs. Only the traits with at least two associated genes were taken into account for calculation of enrichment. Significance of an enrichment of SNPs associated with a trait was calculated using hypergeometric distribution.

The current study significantly improved the overlap of transcriptional signatures with SNPs associated with Th17 cell-mediated diseases as compared to our earlier report applying microarrays (Table 1) [15]. Next, we analyzed whether combination of the expression profiling data with the SNP information, could be used to suggest the importance of Th17 cells in diseases not generally considered as Th17 cell-mediated. Based on the overlap, we found evidence that Th17 cells might play a role in diseases such as celiac disease, hypertension and Parkinson's disease (Figure 4, Table S10). When disease-associated polymorphisms were superimposed to the differentially expressed lncRNAs, we found a significant enrichment of non-coding transcripts harboring SNPs associated e.g. with celiac disease, schizophrenia, systemic lupus erythematosus and Crohn's disease suggesting that lncRNAs might be involved in etiology of these disease (Table 2).

**Table 1:** Enrichment of the SNPs associated with selected Th17 cell-mediated diseases among the differentially expressed genes in the Th17 cell transcriptomics studies

	Array^a^		RNA-seq^b^	
Trait	FDR	No. of genes	FDR	No. of genes
Arthritis, Rheumatoid	5.80E-02	30	2.02E-14	64
Asthma	1.60E-01	36	1.39E-07	69
Dermatitis, Atopic	2-87E-01	4	9.48E-04	10
Inflammatory Bowel Diseases	3.05E-01	12	4.77E-03	21
Multiple Sclerosis	5.36E-01	22	2.06E-05	49
Psoriasis	6.03E-02	24	1.06E-08	42

The indicated diseases are Th17-cell mediated based on Tesmer *et al.* 2008 [49]

a Tuomela *et al*. Blood 2012 [12]

b The current study

**Table 2:** The traits showing the strongest enrichment of differentially regulated lncRNAs in our Th17 cell polarization data

Trait	FDR	No. of lncRNAs
Celiac Disease	1.18E-03	9
Schizophrenia	4.69E-03	16
Azoospermia	4.69E-03	3
Alopecia Areata	4.69E-03	4
Lupus Erythematosus, Systemic	4.69E-03	13
Crohn Disease	5.49E-03	10
Diabetes Mellitus, Type 1	5.61E-03	10
Follicle Stimulating Hormone	1.12E-02	4
Mucocutaneous Lymph Node Syndrome	1.75E-02	4
Personality	2.93E-02	3

When the enrichment of disease-associated SNPs (Table S10) was compared among the top 20% genes which were regulated during human Th17 cell priming (Table S4) or the fraction of the top 20% genes which were regulated similarly between human and mouse (Table S4) the statistical power of the analysis remained significant (Figure 4). Instead, when the top 20% of the genes regulated in mouse (Table S4) was used in the analysis the overlap between the regulated genes and the disease-associated SNPs was in many cases insignificant and substantially lower than among the similarly regulated genes (Figure 4). In conclusion, identification of similarly regulated genes between human and mouse pinpoints signaling pathways predisposing to Th17 cell-mediated diseases and which can be studied with mouse models.

## DISCUSSION

Th17 cells secreting IL17 are crucial for controlling extracellular bacterial and fungal infections, and their presence in small intestine is essential for barrier protection preserving intestinal homeostasis [19]. Wide variety of cell types are responsive for IL17 [20], and thus differentiation and actions of Th17 cells need to be tightly regulated. Improper Th17 cell activity has been associated with several autoimmune diseases and development of cancer [3, 19].

We report a considerable number of novel Th17 cell polarization stimulus responding coding genes, and for the first time the expression of lncRNAs during the initiation of human Th17 cell differentiation starting from cord blood CD4+ cells. Inclusion of the coding genes newly reported to be regulated during Th17 cell priming in this study considerably improved the overlap between the genes and the SNPs associated with Th17 cell-mediated diseases. We also found a significant overlap between the differentially expressed lncRNAs and SNPs associated with disorders such as rheumatoid arthritis, multiple sclerosis and psoriasis suggesting an involvement of non-coding transcripts in the Th17 cell-mediated pathogenesis of these diseases. In addition, our data suggest that activity of Th17 cells plays a role in a wider variety of diseases than currently recognized.

In order to distinguish the genes which are suitable for validation of their therapeutic potential with mouse models and the ones which require alternative approaches in preclinical studies we compared expression profiles of human and mouse cells polarized toward Th17 cell phenotype. Transcriptional signature containing three hundred strongly regulated genes similarly responding to the induction of Th17 cell differentiation in human and mouse was identified. ENCODE consortium reported that the *cis*-regulatory regions of the genes active in the immune system have especially gone through diversification since the common ancestor of human and mouse. However, *trans*-regulation of the genome was found to be more similar between the species than *cis*-regulation suggesting plasticity in the mechanisms of gene expression regulation [21]. Our finding of the importance of the conserved Th17 gene expression signature in highlighting candidate genes based on the overlap with the disease-associated polymorphisms is in line with this observation. The lincRNA profiles of Th cell subtypes have been reported to be more distinct than the mRNA profiles indicating that selective expression of lncRNAs is crucial for the phenotype specification [8, 11]. In human, lncRNAs represent 24% of the whole transcriptome, whereas only 10% in mouse, [22] suggesting that non-coding RNAs might play particularly important role in human. Based on the current knowledge most of the differentially regulated lncRNAs identified in our human samples did not have a counterpart in mouse. However, new tools for predicting conservation for example exploiting modelling of RNA secondary structure are needed.

Mouse models are valuable tools in characterization of signalling pathways and in biomedical research, and most of the studies on Th17 cells have used mouse as a model organism. However, immunological differences between human and mouse are known to exist [23, 24, 25] and translation of findings done with model organisms to human therapeutics has been challenging [26, 27]. Collectively our SNP analysis and the coding and non-coding transcriptome results highlighted the importance of investigating human Th17 cell priming and function to complement the studies with model organisms to improve translation of lab inventions into clinical benefits.

## MATERIALS AND METHODS

### Human CD4+ T-cell isolation and Th17 cell culture

Human mononuclear cells were isolated from the umbilical cord blood of healthy neonates (Turku University Central Hospital, Turku, Finland) using Ficoll-Paque isolation (Ficoll-Paque PLUS; GE Healthcare). CD4+ cells were further purified (Dynal CD4 Positive Isolation Kit; Invitrogen) and after the isolation cells from several individuals were pooled. Cells were activated with plate-bound αCD3 (750 ng/24-well culture plate well; Immunotech) and soluble αCD28 (1 μg/mL; Immunotech) in a density of 0.5 × 10^6^ cells/mL of X-vivo 20 serum-free medium (Lonza). The media was supplemented with 2 mM L-glutamine (Sigma-Aldrich), and 50 U/mL penicillin and 50 μg/mL streptomycin (Sigma-Aldrich). Cells were polarized toward Th17 direction with IL6 (20 ng/mL; Roche), IL1β (10 ng/mL) and TGFβ (10 ng/mL) in the presence of neutralizing anti-IFNγ (1 μg/mL) and anti-IL4 (1 μg/mL). Cells activated without differentiating cytokines but with only neutralizing antibodies were also cultured as controls (Th0). All cytokines and neutralizing antibodies were from R&D Systems unless otherwise stated.

### Mouse *in vitro* CD4+ T cell differentiation

C57BL/6, mice were bred in the NIMR animal facility under specified pathogen free conditions. Naïve CD4 T cells (CD4^+^CD25^−^CD44^lo^) were isolated from C57BL/6 mice and the cells were sort-purified (average purity 99.9.%) with MoFlo flow cytometer (Beckman Coulter) and cultured in IMDM (Sigma-Aldrich) supplemented with 5% FCS, 2×10^−3^ M L-glutamine, 100 U/ml penicillin, 100 μg/ml streptomycin and 5×10^−5^ M β-mercaptoethanol (all Sigma) in the presence of plate-bound αCD3 (0.5 μg/ml; 2C11) and plate-bound αCD28 (5 μg/ml; 37.51; both from Large Scale Facility, Medical Research Council National Institute for Medical Research). Th17 differentiation was induced by culturing the cells in the presence of TGFβ (1 ng/ml), IL6 (20 ng/ml), and IL1β (10 ng/ml) (R&D Systems). Part of the cells was cultured without any polarizing cytokines, in “Th0” conditions.

### RNA-seq sample preparation and preprocessing

Three biological replicates of samples were collected at 0, 0.5, 1, 2, 4, 6, 12, 24, 48, and 72 hours time points. RNA was isolated (RNeasy Mini Kit, QIAGEN) and DNase treated (RNase-Free Dnase Set; QIAGEN). RNA-seq with 50 nt read length was performed at Illumina sequencing service provider with HiSeq 2000 instrument using TruSeq chemistry and the raw data was basecalled with CASAVA1.8. Five of the samples were paired end, with read length 75nt. Those reads were truncated to 50nt and only one of the paired ends was used. Sequence reads were mapped using Tophat (version 1.3.2) with default parameters to the GRCh37 human reference genome, Ensembl human transcriptome (release 63) and GENCODE lncRNA (release 18) annotations for human, and to the NCBIM37 mouse reference genome, Ensembl mouse transcriptome (release 63) and NONCODE (version 3.0) lncRNA annotations for mouse. Expression levels were estimated for Ensembl genes using Python script rpkmforgenes with parameters -readcount -no3utr -rmnameoverlap -bamu [28]. 3′ untranslated regions were ignored and regions where several transcripts with different gene identifiers overlap were removed. Genes with RPKM values <3 in at least two replicates at all time points were filtered out from the downstream analysis.

### Differential expression

Bioconductor package edgeR [29] was used to define differential expression between Th17 and Th0 conditions. Differential expression calling for human samples was performed for each time point taking into account the paired experimental design. For mouse samples there was no paired design between Th0 and Th17 replicates. The dispersion was estimated as gene-wise dispersion. The differentially expressed genes were identified with FDR <0.05 and |log2 FC| >1 cut offs. Using a false discovery rate (FDR) <0.05 and filtering out genes with |log2 FC| <0.3 the differentially expressed genes were ranked based of their log2 FC. Twenty percent of the up and down regulated genes at each time point with the largest |log2 FC| were selected for further analysis in each species. For the comparisons all the time points were merged. Genes were mapped one-to-one between human and mouse using Ensembl Biomart database.

### Clustering

Differentially regulated genes in both human and mouse were clustered together using k-means clustering with k = 30. If orthologous genes were in the same cluster they were considered to have the similar kind of expression profile. Clustering was done for the average standardized log-transformed RPKM-values. Heatmaps of the expression values and fold changes of the similarly regulated human and mouse genes were clustered using hierarchical clustering with Euclidean distances and *Ward's* minimum variance method.

### LIGAP

Gaussian process regression has been used before e.g. for calling differentially expressed genes between two or more treatments in the time course microarray data [16, 30]. We apply and extend a recently proposed LIGAP method [16] which uses non-parametric Gaussian process regression to compare the kinetics of gene expression during early polarization of Th17 cells between orthologous human and mouse genes. We compare two models: Th17 cell profiles of an orthologous gene behave in the same way in mouse and human, or they behave in a different way. In the first model Th17 cell data of both species is described by a single latent non-parametric function. In the second model, we fit two independent models with two Gaussian processes, one for mouse Th17 cell profile and one for human. Expression values are log-transformed and standardized.

We set a Gaussian process prior for the expression values

f(x)~GP(m(x),k(x,x′)),

where *m*(*x*) is the mean function and *K*=*k*(*x*, *x*′) is the neural network covariance function $k(x,\;x\prime )=s_{f}^{2}\;\sin^{-1}\left(\frac{{\tilde{x}}^{T}\text{diag}(l^{-2})\tilde{x}\prime }{\left(1+{\tilde{x}}^{T}\text{diag}(l^{-2})\tilde{x})(1+\tilde{x}\prime^{T}\text{diag}(l^{-2})\tilde{x}\prime \right)}\right),$ where vectors $\tilde{x}$ and $\tilde{x}\prime$ are augmented by unit value, *l* is the length scale and $s_{f}^{2}$ quantifies the amount of signal variance.

We set the mean function *m*(*x*) to 0. The predictions by a Gaussian process are made in the following way

$$p(y_{*}|Y,X,x_{*})=N(\mu_{*},\sigma_{*}^{2}),$$

where Y is the vector of gene expression values at time points X, x_*_ are the new time points where we want to predict expression y_*_ and $\mu_{*}=(k_{X,x_{*}}^{T}{\left(K+\sigma_{n}^{2}I\right)}^{-1})Y,\sigma_{*}^{2}=k(x_{*},x_{*})-k_{X,x_{*}}^{T}{\left(K+\sigma_{n}^{2}I\right)}^{-1}k_{X,x_{*}}.$ *K*=*k*(*X*, *X*) and $\sigma_{n}^{2}$ is the noise variance which follows a Gaussian distribution with zero mean.

In our model we have four hyper parameters: length scale *l,* signal variance $s_{f}^{2}$, variance of white Gaussian noise $\sigma_{n}^{2}$ and the time shift Δ_t_, which tells how much the mouse Th17 cell profile is delayed or ahead compared to human Th17 cell profile [31]. The time shift parameter is added to mouse time points in a shared model. The time shift parameter is restricted between −24 and 24 hours. The hyper parameters of neural network covariance function are optimized by maximizing the marginal likelihood using a conjugate gradient method. To prefer smooth functions, the prior on the length scale is set to *l*∼Γ(6,30). The prior probability of signal variance is $s_{f}^{2}$∼Γ(10,10). The prior of the noise variance is also Gamma distributed. Parameters of the distribution are estimated in the same manner as in Cooke *et al.* 2011 but now the mode for the noise variance is thought to be the average of the variances of replicates and the lower bound is a small number ε and the upper bound is one because of the standardization of expression values. The two alternative models, shared and independent, can be compared using Bayes factor [16, 30].

$$BF=\frac{P(Y_{\text{mouse}}\cup Y_{\text{human}}|H_{\text{GP}},X_{\text{mouse}},X_{\text{human}})}{P(Y_{\text{mouse}}|H_{\text{GP}},X_{\text{mouse}})P(Y_{\text{human}}|H_{\text{GP}},X_{\text{human}})}.$$

The union symbol means that in the shared model data is treated as being generated from a single model. The Bayes factor score over 10 shows evidence for the shared model, in which case the orthologous genes were considered as having the same expression profile.

### LncRNA analysis

Human lncRNAs were obtained from Gencode release 18 (http://www.gencodegenes.org) database and mouse lncRNAs from NONCODE v3 (http://noncode.org). Expression values of lncRNAs were calculated using Python package HTSeq [32], where overlapping reads with protein-coding genes were excluded. LncRNAs with RPKM values <0.5 in at least two replicates at all time points were filtered out from the downstream analysis [33]. Differential expression calling was done using Bioconductor package edgeR [29], as described above. To define orthologous lncRNAs between human and mouse, human lncRNA coordinates were converted to mouse coordinates using the liftOver tool of the UCSC Genome Browser [18]. The minimal overlap between the converted coordinates and the known mouse lncRNAs was set to 100 nucleotides. The differentially expressed protein coding genes having the highest and the lowest correlation with the differentially expressed lncRNAs were calculated using Pearson correlation. Enrichment analysis of the nearest genes and the correlated genes was performed using GeneTrail tool [34].

### Transcription factor motif detection

Identification of transcription factor binding motifs was done with Homer software (http://biowhat.ucsd.edu/homer/ngs/index.html). The binding sites of five transcription factors; BATF, IRF4, STAT3, MAF, and RORC at 48 hours after priming of mouse Th17 cells [13] were used to find the binding motifs at promoter areas of orthologous human and mouse genes and at promoter areas of similarly regulated orthologous human and mouse genes. All the promoter areas of orthologous genes were used as a background. The identification of binding motifs was performed separately for mouse and human promoter regions using only the genes that had a ChIP-seq detected binding site of one of the transcription factors in its promoter area in mouse Th17 cells [13]. Motifs were searched from four different regions relative to the TSS; [−250,250], [−500, 500], [−750, 750], [−1000, 1000]. The binding sites of BATF and IRF4 were divided into three subcategories; BATF only, IRF4 only and BATF+IRF4 only. Composite motif of BATF+IRF4 could not be recovered when analysing only the binding sites at promoters. *De novo* search was performed for the 500 most significant binding sites of BATF and IRF4 and then the composite motif found was used to scan the promoter areas of the similarly regulated genes. The significance of overlap between similarly regulated genes between the two species that had the same binding motif was determined using hypergeometric distribution.

### Co-localization analysis

Co-localization analysis was carried out separately for similarly regulated human and mouse genes using Kerfuffle gene co-localization analysis tool [35] with parameters d <=3 and lowest *p*-value =1e-2. The *p*-value for each cluster was determined by randomly distributing the genes across the genome and by calculating the distances between the genes. The distances between the real positions and random positions were compared and the *p*-value is the frequency that randomly permutated cluster counts exceeds the number of the real clusters.

### SNP analysis

Disease-associated lead SNPs were obtained from NCBI database (http://www.ncbi.nlm.nih.gov/projects/gapplusprev/sgap_plus.htm). SNPs which were associated to a certain disease with *p*-value <1e-5 were included in the analysis. Genes linked to a SNP were determined with a +/−100 kb window. Analysis was done for the different gene sets; shared Th17 specific genes between human and mouse, and human and mouse top 20 % genes. Among top 20% regulated mouse genes, only orthologous genes were considered and SNPs were linked to mouse genes *via* the orthologous human genes. Diseases with less than two associated genes were excluded in the enrichment analysis. Significance of an enrichment of a trait was calculated using hypergeometric distribution. For the common genes and the mouse genes all orthologous genes were used as a reference set. For human genes the reference set was all the human genes.

### Gene set enrichment analysis

Time point-specific expression data (RPKM values) was ranked using signal-to-noise ratio metric followed by analysis of enrichment of pathways in the ranked list [36]. Gene sets were pathways from REACTOME database (http://www.reactome.org). The FDR cut off for the included pathways was 0.05.

### Functional enrichment analysis

Functional classes for human and mouse genes were obtained from IPA (http://www.ingenuity.com/, September 2013) for genes annotated as “cytokine”, “G-protein coupled receptor”, “growth factor”, “ion channel”, “kinase”, “ligand-dependent nuclear receptor”, “mature microRNA”, “microRNA”, “peptidase”, “phosphatase”, “transcription regulator”, “translation regulator”, “transmembrane receptor”, or “transporter”. Significance of an enrichment of a functional class among the top 20% differentially regulated genes in both species was calculated using hypergeometric distribution, where the reference set of genes was all the human or mouse genes.

### Ethical aspects

The usage of blood of unknown donors was approved by the Ethics Committee of the Hospital District of Southwest Finland. Animal experiments were done according to NIMR institutional guidelines and Home Office regulations.

### Accession numbers

The data discussed in this publication are accessible through GEO Series accession number GSE52260 (http://www.ncbi.nlm.nih.gov/geo/query/acc.cgi?acc=GSE52260).

## SUPPLEMENTARY MATERIAL FIGURES AND TABLES






















