# Supplementary material for: Comparative analysis of human and mouse transcriptomes of Th17 cell priming
Source: Oncotarget. 2016 Mar 7;7(12):13416–28. doi: 10.18632/oncotarget.7963 (PMC4924651; doi:10.18632/oncotarget.7963)
Supplement: Supplementary file 1 [file oncotarget-07-13416-s001.pdf]

Comparative analysis of human and mouse transcriptomes of Th17 cell priming

Supplementary Material

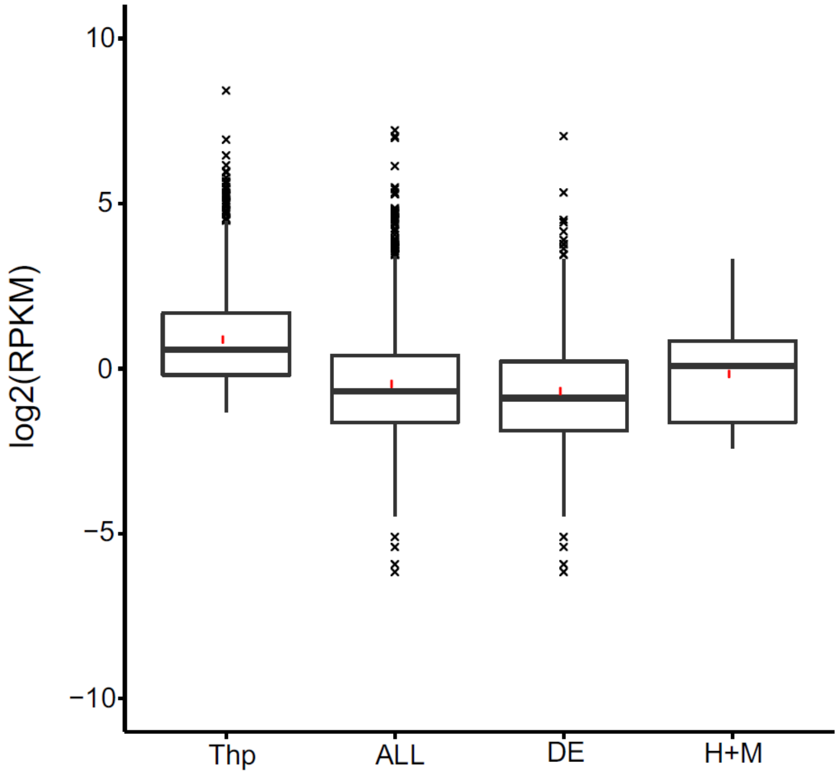

Fig. S1. Expression level of lncRNAs expressed during Th17 cell polarization. The distribution of the expression levels (log2 of the RPKM values) of lncRNAs expressed before activation (Thp) or after activation (ALL), the differentially regulated lncRNAs (DE), and the differentially regulated lncRNAs shared between human and mouse (H+M) during human Th17 cell priming. The average of expression values are shown with red color

A

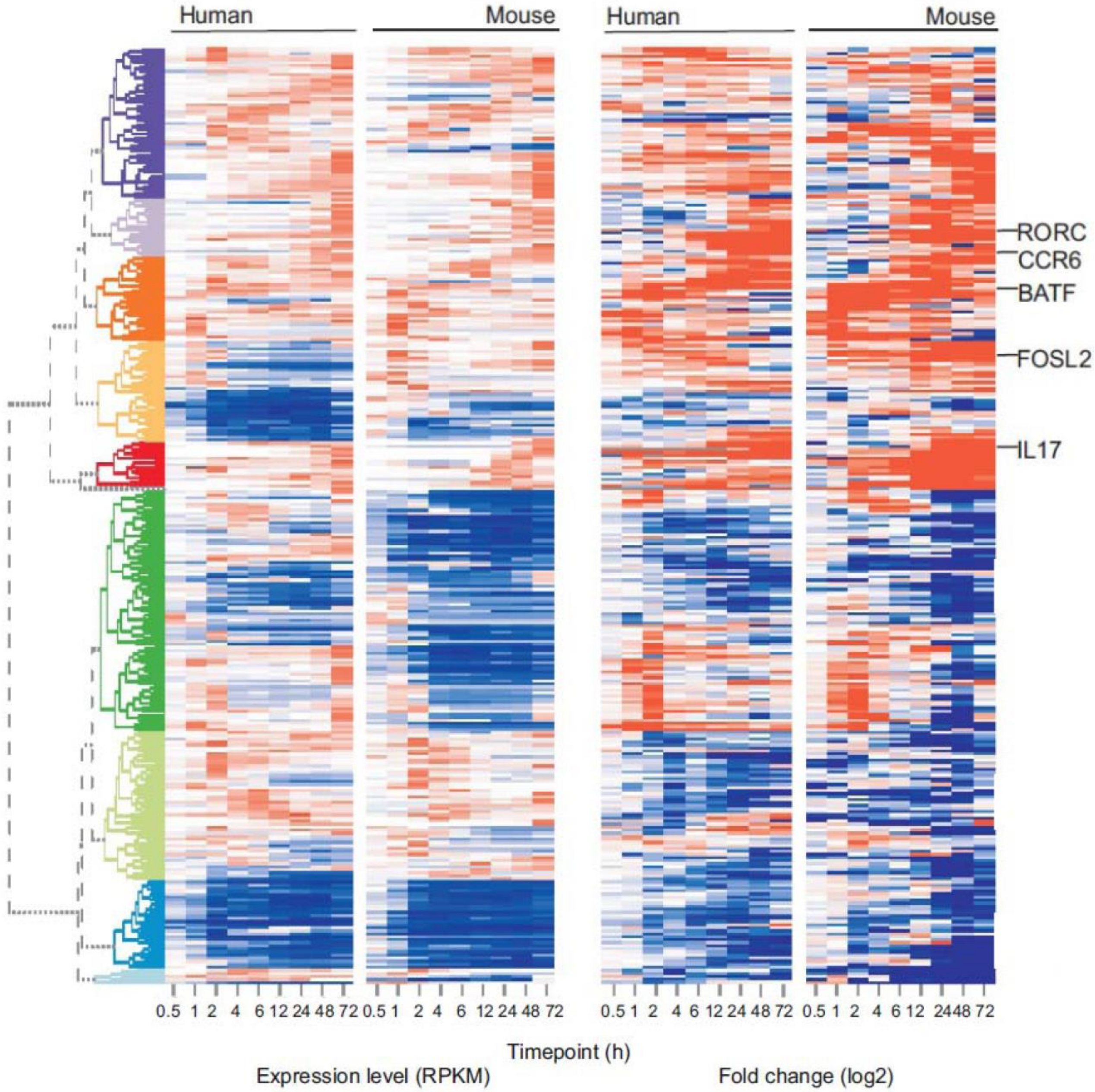

Human
Mouse
Human
Mouse
RORC
CCR6
BATF
FOSL2
IL17
0.5 1 2 4 6 12 24 48 72
0.5 1 2 4 6 12 24 48 72
0.5 1 2 4 6 12 24 48 72
0.5 1 2 4 6 12 24 48 72
Timepoint (h)
Expression level (RPKM)
Fold change (log2)

B

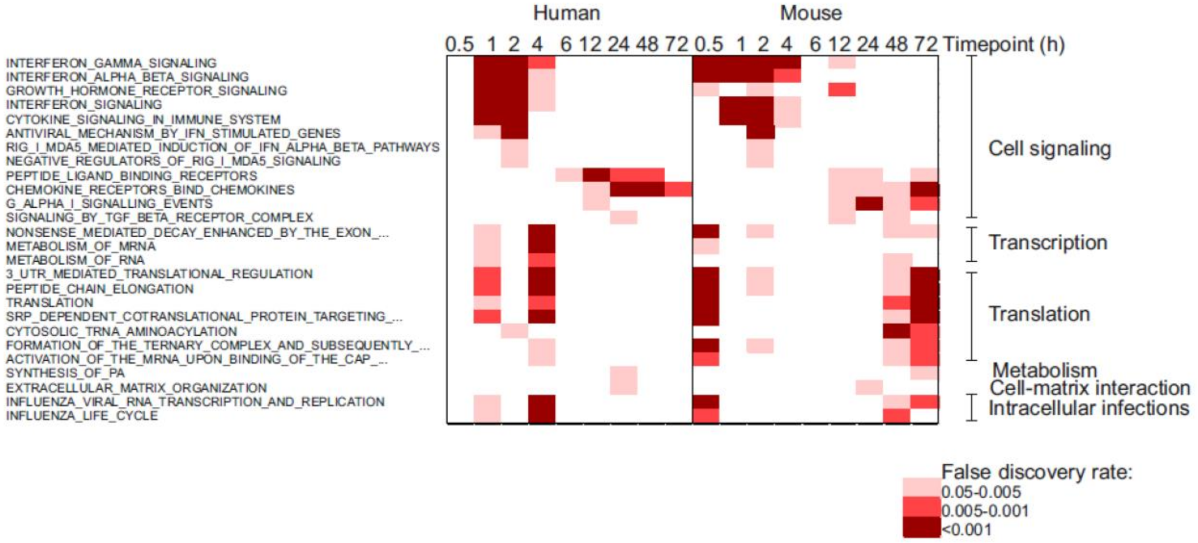

Fig. S2. Shared Th17 cell-specific transcriptome in human and mouse. (A) Genes with FDR <0.05 were ranked based on their fold change between Th17 and Th0 conditions. The top 20% of the ranked up-regulated and down-regulated orthologous genes were included in the analysis. The similarly regulated genes had conserved regulation at least at one time point during the analyzed time frame. The shared Th17 cell-specific genes in human and mouse are presented as a hierarchically clustered heat map of the z-normalized expression levels (RPKM-values) in Th17 cells (left) and log2 fold changes between Th17 and Th0 cells (right). The genes up-regulated in Th17 condition are marked in red and the down-regulated in blue. Selected Th17 cell marker genes are highlighted in the figure. The clustering was performed using RPKM and log2 FC values of human and mouse genes. See Table S4 for the full results. (B) Gene Set Enrichment Analysis (GSEA, Broad Institute) of the time point-specific enrichment of regulated pathways in human and mouse. The significant pathways (FDR <0.05) common in both human and mouse are presented with color code representing the indicated significance levels. Names of some of the pathways were truncated for clarity. See Table S6 for the full results.

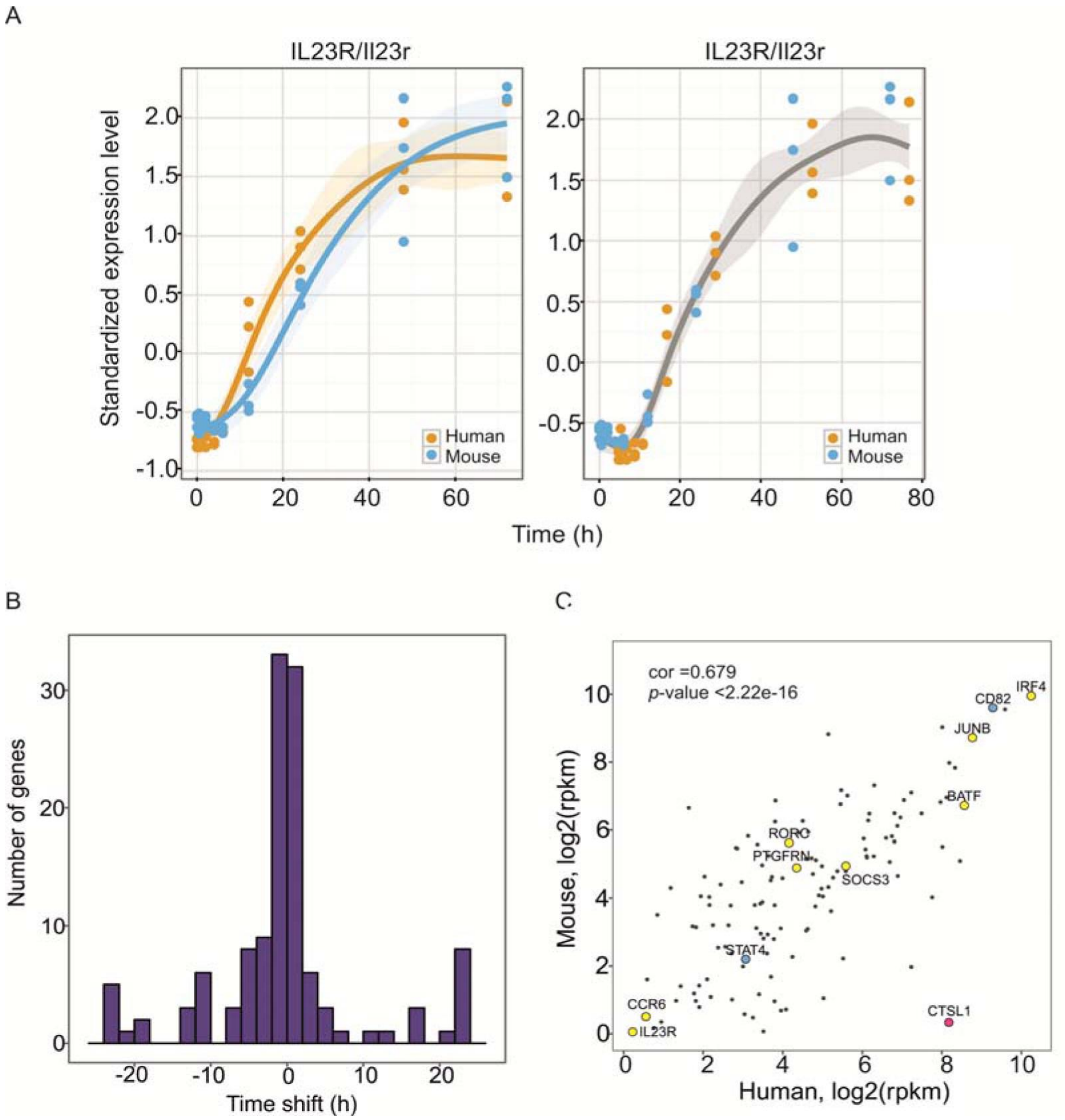

Fig. S3. Conserved Th17 cell gene expression patterns between human and mouse. (A) Functionality of the extended LIGAP method using *IL23R* as an example. Two alternative models; independent (left) and shared (right) are shown. Independent model is described by separate latent non-parametric functions for human and mouse whereas a shared model is described by a single function. The yellow and blue dots indicate expression in each human and mouse sample,

respectively, and the solid gray line the calculated average expression profile with the shaded 95% confidence interval. (B) The distribution of the time shifts of transcriptional regulation detected with the extended LIGAP method. Positive value indicates a delay in the expression in mouse compared to the orthologous human gene, and a negative value indicates that the mouse gene is expressed earlier than the human gene. The maximum time difference was fixed to 24 hours. (C) Correlation of the gene expression levels in Th17 cell polarizing conditions (log2 of the RPMK values) for the genes with a similar gene expression pattern in Th17 cells in human and mouse was evaluated with the extended LIGAP time shift modeling. The correlation data at 6 hours is plotted as an example with Pearson correlation coefficient and p-value. Selected Th17 cell marker genes, STAT4 down-regulated in Th17 cells and CTSL1 validated to be up-regulated (Tuomela et al., 2012) in human Th17 cells are highlighted in the figure with yellow, blue and pink, respectively. The expression level correlations at all detection timepoint are listed in Table S7.

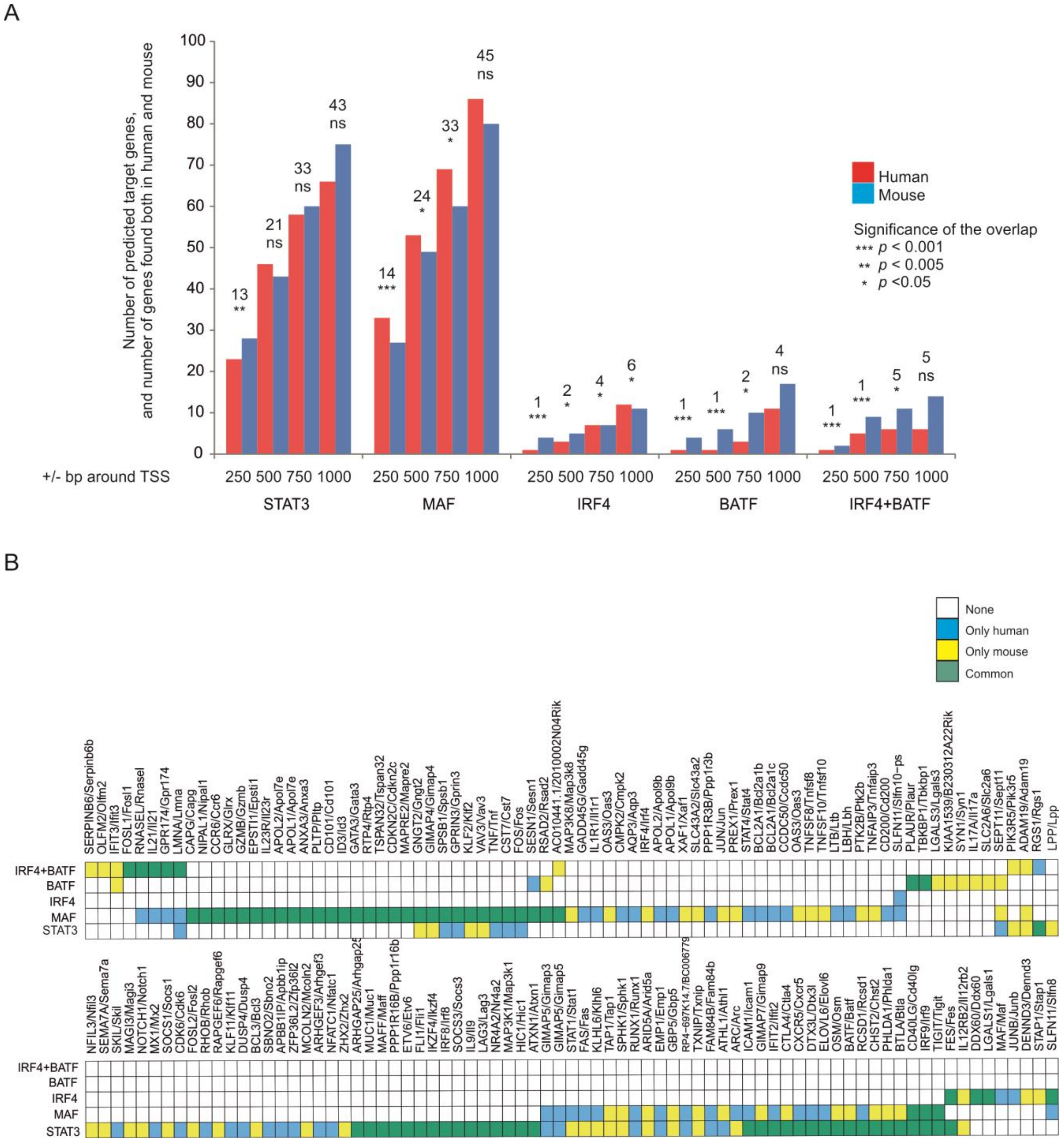

Fig. S4. Transcriptional circuitry in Th17 cells. Global DNA binding pattern of BATF, IRF4, STAT3, MAF, and RORC at 48 hours after initiation of mouse Th17 cell differentiation (Ciofani et al., 2012) was overlaid to our data to search for promoter proximal DNA binding motifs of these factors among the top 20% shared orthologs between human and mouse with analysis windows of

+/-250, 500, 750 and 1000 bp around the transcription start sites (TSS). (A) The number of genes predicted to be bound by STAT3, MAF, IRF4, BATF or IRF4 and BATF together. The number of the similarly regulated genes between human and mouse that have a same binding motif is indicated above the bars with the statistical significance of the overlap. (B) Predicted target genes with +/-750 bp window around the TSSs in human and mouse.

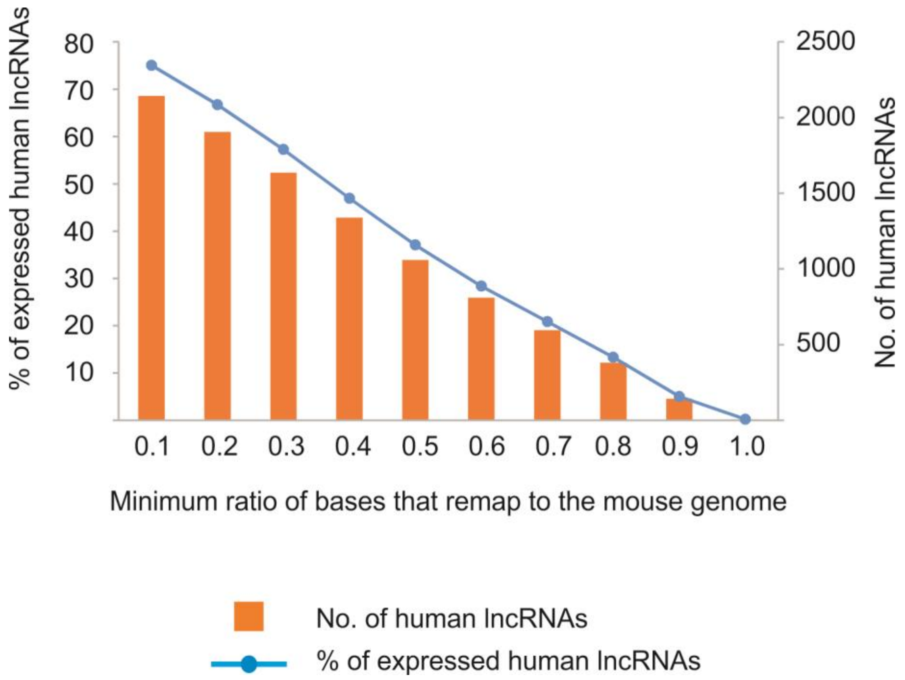

Fig. S5. Conservation of the human lncRNA sequences in the mouse genome. Sequence level analysis of the conservation of the lncRNAs expressed during human Th17 cell polarization in the mouse genome. The blue line with dots and the orange bars represents the percentage and the number of the expressed lncRNAs in human, respectively. The ratio of bases of the expressed human lncRNA that can be remapped to the mouse genome is presented on x-axis.
